# Young People’s Mental Health

**DOI:** 10.1007/s43151-022-00079-3

**Published:** 2022-07-04

**Authors:** Johanna Wyn

**Affiliations:** Melbourne Graduate School of Education, The University of Melbourne, Victoria 3010 Parkville, Australia

**Keywords:** Youth, Mental health, Wellbeing, Resilience

The issue of young people’s mental health is receiving increasing attention in youth studies, responding to the high incidence of mental health issues amongst the young population. Against this backdrop is timely to reflect on youth studies approaches to the issue of young people’s mental health and how it makes to a contribution to a topic that has, until recently, been almost universally regarded as the province of the psy-knowledges and medical disciplines.

The evidence that a significant proportion of young people experience poor mental health and that this has worsened over time is clear enough. Fifteen percent of young Australians aged 18–24 experienced high or very high levels of psychological distress in 2017–2018 (Australian Institute of Health and Welfare 2021), and this figure seems set to rise. Mission Australia and the Black Dog Institute found that young Australians aged 15–19 years experienced an increase in psychological distress, from 18.6% in 2012 to 26.6% in 2020 (Brennan et al. 2021). Other research concludes that in Australia, “mental health issues have increased, with young people carrying a disproportionate burden” (Einboden et al. 2020: 355). Research on the effects of the COVID-19 pandemic on young people’s mental health has intensified interest in this issue. An online survey administered to a sample of 760 Australian adolescents aged 12–18 years in 2021 found that three quarters of the sample experienced a worsening in mental health since the pandemic began, impacting negatively on their learning, friendships and family relationships and bringing increased levels of sleep disturbance, psychological distress and health anxiety (Li et al. 2021).

The heightened interest in young people’s mental health is reflected in a scan of recent articles in journals focused on youth studies, which reveal a raft of research that explores the association between social conditions and poor mental health. Examples include research focusing on groups of young people who are disadvantaged, subject to bullying or other forms of violence or are marginalised, but not all focus on typically “vulnerable” young people. Some focus on places of work or study. The scan included articles on apprentices’ relationships in workplaces (Einboden et al. 2020); the link between agency, resistance and inequality for young women in regional communities (te Riele and Shelley 2021); struggles for belonging and recognition for young people who identify as LGBTQ + (Wike et al. 2021); the challenges of striving for educational goals and body ideals (Eriksen 2021); and experiences of shame and anger in school settings (Moensted 2022).

Many of these articles epitomise the tightrope researchers and practitioners in the youth mental health space walk in avoiding the tendency to individualise mental health as a form of personal vulnerability and responsibility, while at the same time recognising and addressing the immediate circumstances of those who are struggling, as well as the social conditions that are associated with this struggle. Creating the right balance between the psychological and social terrain of mental health has been explicitly explored in the wider field of the sociology of health and illness and bears a closer look. For example, focusing on the related issue of suicide, Fitzpatrick (2022: 113) draws attention to the “web of social, moral and political relations” within which suicide occurs, seeking to make these relations explicit through a framework of “moral and political economy.” Using the concept of the moral economy, Fitzpatrick shows how identifying “vulnerable individuals and groups to improve service and resource provision, the identification, assessment, monitoring and reduction of risk have become an integral part of professional and community action and judgement” (2022: 118). An unintended effect of this development, Fitzpatrick argues, is that the containment of risk overrides “care practices that acknowledge human agency and the dignity of risk and choice” (2022: 118–9) and positions the individual (solely) as the locus of interventions. The focus on individuals, he argues, emphasises social obligations and personal responsibilities to be well by those who are not well, “through education programs that target mental health literacy, help-seeking, stress management, resilience, problem solving and coping skills” (Fitzpatrick 2022: 119). The pressure on individuals to take responsibility for their wellbeing, Fitzpatrick argues, is bound to “an economy of moral values and norms” that defines “morally good or bad actions and practices” (2022: 120) and obscures dissenting views which offer an alternative to medicalised approaches to mental health. Alternative approaches include recognition of the social determinants of wellbeing, including “the dispossession of First Nations people, economic adversity, environmental conditions, and other social issues” (Fitzpatrick 2022: 120). The point I take from his work is that the issue of young people’s mental health: how it is defined, and how it is treated, is framed by a political and moral economy that needs to be made explicit.

A similar point about mental health was made previously by Ecclestone (2012) who argues for a reconsideration of C. W. Mills’ sociological imagination in relation to mental health, highlighting the importance of understanding the historical emergence of particular ways of viewing human nature. She notes the tendency for social science to (one the one hand) recognise the role played by the “realities and complexities of modern life” in people’s mental health, and yet (on the other hand) to shift wellbeing to “to a psychological terrain … that emerges from, and reinforces, a cultural therapeutic ethos rooted in determinist assumptions about emotional and psychological vulnerability” (2012: 476).

McLeod and Wright take this challenge directly to youth studies. They bring a critical lens to the field of young people’s wellbeing to raise debate about “its transformative promise as well as its individualizing effects” (2016: 776). Asking “what does wellbeing do?”, they explore the unintended effects of policy discourses about young people’s mental health. Their analysis provides an appreciation of the slippery nature of these discourses in youth studies research and programs in schools. It highlights the historical shift from mental health interventions targeted at remediating specific groups of young people who have been diagnosed as having mental health problems or who are deemed at risk to a contemporary approach which favours universal programs that have a preventive goal. They argue, drawing on Cigman (2012), that a critical lens is needed to guard against the potential for universal approaches to cast particular “emotional states and subjective dispositions as either positive or negative” a limiting binary that they suggest reflects the influence of positive psychology on thinking about wellbeing (McLeod and Wright 2016: 788). In this, McLeod and Wright foreshadow Fitzgerald’s concept of the “moral economy” of mental health referred to above. They conclude that without a critical approach to the concepts of mental health and wellbeing, individualising conceptions can creep in, to obscure the social justice and equity dimensions that are fundamental to young people’s mental health. These individualising conceptions align with neoliberal approaches that emphasise individual responsibility for managing the risks that conditions impose on young people.

A closer look at the way in which mental health is being approached in the field of youth studies suggests that these criticisms and cautions are being heard. Each of the articles referred to in the above scan of the *youth studies journals* recognises the immediate situation of young people while locating them within the social and political milieu which gives rise to poor mental health. While the identification of circumstances (such as social or economic disadvantage, violence, or marginalisation) is a nod to a “social determinist” approach, these authors take steps beyond the identification of social determinants, to provide a relational analysis that reveals the complex dynamics between individuals, institutions and policies that influence mental health. For example, Einboden et al. (2020) explore how relationships in workplaces impact negatively on young apprentices, identifying the conditions of long work hours, job insecurity, low wages poor quality education and training, bullying and an unresponsive hierarchy of authority and unrealistic expectations for their performance create an ongoing situation that is stressful, frustrating and debilitating. They conclude that the poor mental health of the Australian apprentices they studied were related to “systemic issues in relation to the conditions of apprentices’ workplaces that warrant reform” Einboden et al. (2020: 365–6). Similarly, Wike et al. (2021) explore the nature and quality of relationships within which young people who identify as LGBTQ + are embedded to show how “thwarted belongingness” exacerbates anxiety and depression. Their analysis, which reaches beyond the tendency to locate “vulnerabilities” within individuals, concludes that longitudinal research may offer deeper insights into the impact of social relationships on the mental health of young people.

It is perhaps through this capacity to recognise and move beyond the legacy of the psy-knowledges that youth studies can make its most significant contribution to understanding and addressing young people’s mental health. The concept of mental health is a term that in practice stands for a wide range of conditions (such as anxiety, depression and psychoses) that are complex and impact with often devastating consequences on individual young people and their families. In this sense, mental health is experienced as an individual condition, treated by professionals who are, too often, thinly spread. The understanding that some of these conditions are effects of the wider milieu in which young people are living, and that this wider milieu is where intervention can make a positive difference, requires a shift of focus.

One of the ways that youth studies researchers do this is by recognising the individualising pull of the very concept of “mental health”, turning instead to other concepts that provide greater traction on the dynamics of relationships between individuals and the milieu in which they are living. This is evident in the “rebranding” of the concepts of wellbeing and resilience using a sociological perspective to bring the moral and political economy of health and wellbeing into the picture.

In the interests of brevity, I focus on resilience to illustrate this point (noting existing work on building a sociological concept of wellbeing including McLeod and Wright (2016) and Wyn (2009). As Cahill (forthcoming) points out, resilience is a metaphor used in a range of disciplines including sociology, psychology, ecology, environmental science and engineering. She shows how the binary between a (desirable) state of stability and an (undesirable) state of instability is central to resilience as a characteristic of building materials, a binary that is echoed in psychological conceptions of resilience as a capacity, possessed by individuals to “bounce back” to a stable (normal, healthy) state. To counter this binary, Cahill turns to social and ecological understandings of resilience as the circumstances in which individuals, groups and communities manage instability. Instead, she argues that a sociological concept of resilience can be used to make visible the variety of labour that young people do to navigate both individual and societal transitions. Resilience is reframed to explore the “aesthetics of life-making in the everyday” (Cahill and Leccardi 2020: 67) to bring an expanded framework for considering young people’s lives, highlighting how work that young people do to hold their lives together necessarily spans economic, social and political domains of their lives, resisting the traditional (moral) binary between subjective dispositions as either positive or negative and that between stability and instability. This approach to the concept of resilience makes young people’s subjectivities a key analytical tool for understanding the continuous work they do to be well as they draw on the discourses and storylines available to them. Both social and ecological understandings of resilience eschew the idea that stability is “natural”, focusing instead on the dynamic and shifting nature of relationships that sustain life, resisting the temptation to see the past as a reference point of a stable state that is projected onto the future.

This understanding of resilience focuses on the way in which young people make sense of their experiences and those of their generation, and the strategies they use to live well, to bring insights into the relationships between individuals and the wider social, economic, political and environmental systems in which they live. It “repoliticises” mental health by revealing the specific social conditions, such as poverty, alienation, unemployment, bullying, injustice and violence that characterise “unresilient” communities and institutions in which poor mental health is fostered and exacerbated. Resilience, from this point of view, is constituted by the webs of relationships, resources and dynamics that are enabling or disabling of good mental health for individuals.

A second way in which youth studies brings a shift of focus on mental health is by recognising and advocating for young people’s capacity to identify these challenges and address them. A methodology for doing this, drawing on ecological models (Bonfenbrenner 1979), is illustrated by Cahill et al. (2013), who designed and delivered the *NewGen Asia* program that built capacity for young people affected by HIV in the Asia–Pacific region to be leaders in creating policy changes. This program supported young people to identify the levers for improving the health and wellbeing of young people across “the micro settings of home, school, and community, the institutional settings of the education, health and justice systems; as well as the broader macro level encompassing culture, religion, the economy and the media” (Cahill et al. 2013: 11).

Youth studies have a significant contribution to make to addressing the observation that poor mental health amongst young people is on the rise. By making visible young people’s everyday struggles to be and live well, youth research challenges the notion that the mentally “healthy” simply bounce back from distress. Further, by rendering discernible the marginalisation of young people from economic and housing security, youth research exposes the erosion of the social and economic fabric resources that contribute to or hinder young people’s possibilities to be well (Wyn and Fu forthcoming; Bessant et al. 2017; Woodman and Wyn 2015). In these times of heightened uncertainty, youth researchers have a contribution to make by demonstrating how young people possess the knowledge, will and capability to address the political, economic and environmental challenges to the mental and physical wellbeing on a global scale and to inform policies that go beyond individual resilience and wellbeing to embed responsibility for young people’s mental health in measures that strengthen their access to employment and housing security, affordable education and protection from violence.
